# Use of convolutional neural networks in skin lesion analysis using real world image and non-image data

**DOI:** 10.3389/fmed.2022.946937

**Published:** 2022-10-19

**Authors:** Samantha C. Wong, William Ratliff, Meng Xia, Christine Park, Mark Sendak, Suresh Balu, Ricardo Henao, Lawrence Carin, Meenal K. Kheterpal

**Affiliations:** ^1^Department of Dermatology, Duke University Medical Center, Durham, NC, United States; ^2^Duke Institute for Health Innovation, Duke University, Durham, NC, United States; ^3^Department of Biostatistics and Bioinformatics, Duke University, Durham, NC, United States; ^4^Department of Electrical and Computer Engineering, Duke University, Durham, NC, United States

**Keywords:** convolutional neural network, skin lesion, real world image, clinical decision support, model

## Abstract

**Background:**

Understanding performance of convolutional neural networks (CNNs) for binary (benign vs. malignant) lesion classification based on real world images is important for developing a meaningful clinical decision support (CDS) tool.

**Methods:**

We developed a CNN based on real world smartphone images with histopathological ground truth and tested the utility of structured electronic health record (EHR) data on model performance. Model accuracy was compared against three board-certified dermatologists for clinical validity.

**Results:**

At a classification threshold of 0.5, the sensitivity was 79 vs. 77 vs. 72%, and specificity was 64 vs. 65 vs. 57% for image-alone vs. combined image and clinical data vs. clinical data-alone models, respectively. The PPV was 68 vs. 69 vs. 62%, AUC was 0.79 vs. 0.79 vs. 0.69, and AP was 0.78 vs. 0.79 vs. 0.64 for image-alone vs. combined data vs. clinical data-alone models. Older age, male sex, and number of prior dermatology visits were important positive predictors for malignancy in the clinical data-alone model.

**Conclusion:**

Additional clinical data did not significantly improve CNN image model performance. Model accuracy for predicting malignant lesions was comparable to dermatologists (model: 71.31% vs. 3 dermatologists: 77.87, 69.88, and 71.93%), validating clinical utility. Prospective validation of the model in primary care setting will enhance understanding of the model’s clinical utility.

## Introduction

Access to dermatology appointments is challenging due to both a limited supply of dermatology providers, especially in rural areas (1), and increasing referrals to dermatology (2). According to a survey of dermatologists, the mean ± standard deviation waiting time was 33 ± 32 days, with 64% of the appointments exceeding the criterion cutoff of 3 weeks for new patients and 63% of the appointments exceeding the criterion cutoff of 2 weeks for established patients. Visits for high-risk cases, such as changing pigmented lesions, could even be delayed as long as 38 to 45 days (3). Therefore, skin lesions are often first detected by primary care physicians (P). Up to one third of primary care visits contend with at least one skin problem, with skin tumors being the most common reason for dermatology referral (4). Limited access to dermatology becomes especially concerning when one accounts for the growing Medicare population, which is expected to account for 1 in 5 patients by 2030 due to this population’s higher incidence of skin cancer (5). There has also been an increasing incidence in skin cancers, particularly a 3-fold increase in melanoma incidence, over the past 40 years (6).

Population based skin screenings (ex. SPOTme screenings), skin cancer awareness campaigns and technology-based solutions (ex. smartphone mole mapping applications) have all attempted to improve access to dermatology, none mimicking clinical workflow. To improve the quality of care and contain costs, screening and risk-stratification tools that provide guidance in real time can be embedded into PCP workflows (7). At baseline, P have variable experience and training in dermatology (4) leading to incorrect clinical diagnoses in 56% of cases when compared to histopathology (8). A validated clinical decision support (CDS) system has the potential to help mitigate this variability. Such a tool can also be used to aid successful tele-dermatology workflows that have emerged during the global pandemic (9, 10).

Deep learning algorithms, such as convolutional neural networks, have been developed to classify skin lesions (11, 12). Dermoscopy-based machine learning (ML) algorithms have reached sensitivities of 87.6% (95% CI: 72.72-100.0) and specificities of 83.5% (95% CI: 60.92-100.0) for melanoma diagnosis (13). Classification of squamous cell carcinoma (SCC) and basal cell carcinomas (BCC) with larger datasets improves performance (11, 14). Comparative studies (12, 15) show deep learning models can also perform similarly to dermatologists and superiorly to P and nurse practitioners (16).

However, several issues need attention while assessing the utility of CNN-based models in the primary care setting. Many of these models were developed with high-quality clinical and dermoscopy photographs with limited skin variability from curated image databases, such as the ISIC Archive (dermoscopy), Asan dataset, Hallym dataset, MED-NODE, and the Edinburgh dataset (14). Dermoscopy images are generally of good quality and high resolution with minimal background noise compared to clinical smartphone images, which encompass a wider field and often have lower quality and resolution. Thus, these models may underperform in resource-limited primary care settings lacking dermatoscopes and high-resolution cameras. For wider utilization, smartphone-based imaging is a more promising image capture platform, offering several advantages such as portability, cost-effectiveness, and connectivity to electronic medical records for secure image transfer and storage. With the development of dermatoscopes that can be attached to smartphones, PCPs gain increased access to being able to take better-quality dermatologic images. Therefore, a ML-based CDS tool that is trained, validated, and tested on clinical and dermoscopy images taken with smartphone cameras can democratize screening and triage in the primary care setting with high usability and validity.

Another issue among CNN-based models is missing clinical context when they are tasked with classification, such as incorporating relevant patient demographics and clinical characteristics. Clinical context has been shown to be relevant by Wang et al., who developed a CNN model trained solely on non-image data and medical records, such as history of precancerous lesions and use of certain photosensitizing medications, to predict the development of non-melanoma skin cancer (17). Other models demonstrated that the context of clinical data is useful for human diagnostic prediction, changing the pretest probability of clinical evaluation (12), as well as that the addition of clinical data improved model performance by 3% (16) both during training and evaluation. However, it is unclear if this effect was noted equally in lesion and rash cases, given that majority of top-10 clinical metadata were relevant to rash-specific differential diagnoses (16). Akin to clinical settings, as a patient’s medical history and physical exam are utilized into a working diagnosis, there may be high utility in developing a CNN model that combines both clinical and image data to form a complete working prediction of an individual’s risk of skin cancer.

**FIGURE 1:**
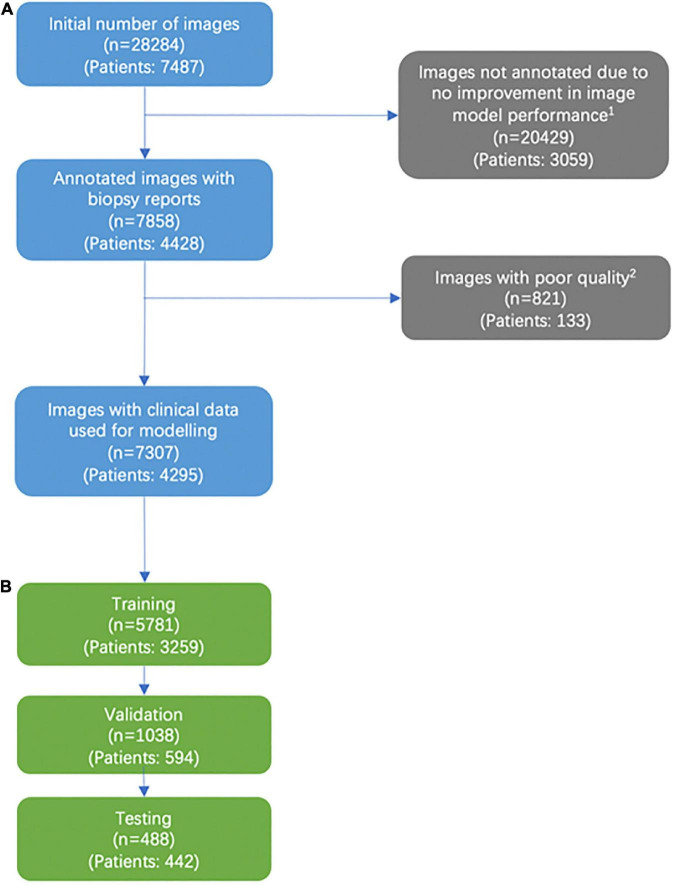
Consort diagram of image selection. **(A)** Addition of labeled images past the 5,141 images that were labeled were found to not significantly improve model performance. **(B)** We excluded images that were deemed not helpful for training our models based on a variety of factors, including images deemed of unusable quality (very low pixels for the area of interest, poor light exposure) and images with predominantly rash diagnosis.

In this study, we developed and evaluated CNN-based models trained on smartphone based clinical and dermoscopy images with histological ground truth, with and without structured EHR clinical data (Figure 1). The models were tasked with classification of skin lesions and tested against board certified dermatologists to test clinical validity. Previous work delineated in a complementary technical paper describing in detail a two staged approach. Because the performance of using a detection model + classification model is better than directly using Faster-RCNN (additional details in technical paper (arXiv:2104.02652, Figures 3A,B), “Malignancy” and “sub-type” are, respectively, two-classes (malignancy and benign) Faster-RCNN and 8-classes Faster-RCNN; the “one-class” is our two stage model. Additionally, Xia et al. show that addition of higher quality images to the model training improves the classification of the model overall, suggesting that the model learns additional features from the higher quality dermoscopy images to better classify clinical images.

In contrast, this paper highlights clinically-relevant aspects of model performance. We describe image selection for training, specific clinicodemographic features selection for training the clinical data-alone and combined models, and the significance of each of the clinicodemographic features in prediction of skin malignancy. This paper also reports the models’ performance against three board-certified dermatologists.

## Materials and methods

We developed a two-stage approach to detect skin lesions of interest in wide-field images taken from consumer grade smartphones. The wide field images underwent an area of interest identification task by the model, followed by binary lesion classification into two groups, malignant vs. benign, for all skin cancers (melanoma, BCC, and SCC) and most common benign tumors. Ground truth malignancy was ascertained *via* biopsy and pathology report. We also sought to investigate model performance for an image-alone model, a structured EHR clinical data-alone model, and a combined model, to see if the addition of clinical data affected the image-alone model’s performance. We looked at 69 discrete clinicodemographic data, with a subset of types represented in Table 1. Statistical significance between bening and malignant groups was estimated *via t*-test and chi-square test for continuous and discrete covariates, respectively. Lastly, we compared the results of the best-performing model against three board-certified dermatologists with different levels of expertise for clinical validation in an office setting.

**TABLE 1:** Demographic and clinical data used in training.

Characteristic	Overall	Benign	Malignant
Age, years (mean ± STD)	61.463 ± 16.285	54.696 ± 17.623	66.535 ± 13.083
**Sex (%)**			
Female	3,504 (48.69)	1766 (57.28)	1738 (42.26)
Male	3,692 (51.31)	1317 (42.72)	2375 (57.74)
**Race^1^ (%)**			
Caucasian/White	6,754 (95.64)	2,783 (92.12)	3,971 (98.27)
Black/African American	152 (2.15)	136 (4.50)	16 (0.40)
Asian	37 (0.52)	31 (1.03)	6 (0.15)
Other	100 (1.42)	56 (1.85)	44 (1.09)
American Indian or Alaskan Native Hawaiian or other Pacific Islander or Not Reported/Declined	19 (0.27)	15 (0.50)	4 (0.10)
**Comorbidities, history of^2^ (%)**			
Skin Disorders	1,057 (14.69)	370 (12.00)	687 (16.70)
No Skin Disorders	6,139 (85.31)	2,713 (88.00)	3,426 (83.30)
Hematological Disorders	225 (3.13)	67 (2.17)	158 (3.84)
No Hematological Disorders	6,971 (96.87)	3,016 (97.83)	3955 (96.16)
Infectious Diseases	76 (1.06)	15 (0.49)	61 (1.48)
No Infectious Diseases	7,120 (98.94)	3,068 (99.51)	4052 (98.52)
Autoimmune Diseases	1,234 (17.15)	450 (14.60)	784 (19.06)
No Autoimmune Diseases	5,962 (82.85)	2,633 (85.40)	3329 (80.94)
**Surgeries, history of^3^ (%)**			
History of transplant	46 (0.64)	20 (0.65)	26 (0.63)
No history of transplant	7,150 (99.36)	3,063 (99.35)	4,087 (99.70)
**Medication use, history of^4^ (%)**			
Oncotherapeutic agents (overall)	1,024 (14.23)	274 (8.89)	750 (18.23)
High risk oncotherapeutic agents	140 (2.08)	30 (4.06)	308 (8.15)
Low risk oncotherapeutic agents	428 (6.35)	120 (3.03)	110 (7.39)
High frequency (≥ 10 administrations)	592 (8.23)	127 (4.12)	465 (11.31)
Low frequency (< 10 administrations)	662 (9.20)	209 (6.78)	453 (11.01)
Did not take oncotherapeutic agents	6,172 (85.77)	2,809 (91.11)	3,363 (81.77)
Immunosuppressants (overall)	2,201 (30.59)	796 (25.82)	1405 (34.16)
High risk immunosuppressants	1,422 (21.49)	538 (18.57)	884 (23.76)
Low risk immunosuppressants	201 (3.04)	72 (2.49)	129 (3.47)
High frequency (≥10 administrations)	438 (6.09)	95 (3.08)	343 (8.34)
Low frequency (<10 administrations)	1,364 (18.95)	553 (17.94)	811 (19.72)
Did not take immunosuppressants	4,995 (69.41)	2,287 (74.18)	2,708 (65.84)
Antibiotics (overall) - all high risk	2,592 (36.02)	953 (30.91)	1,639 (39.85)
High frequency (≥10 administrations)	573 (7.96)	129 (4.18)	444 (10.80)
Low frequency (<administrations)	1,313 (18.25)	538 (17.45)	77 (18.84)
Did not take antibiotics	4,604 (63.98)	2130 (69.09)	2,474 (60.15)
Cardiovascular drugs (overall)	2,573 (35.76)	864 (28.02)	1,709 (41.55)
High risk cardiovascular drugs	35 (0.59)	22 (0.80)	13 (0.40)
Low risk cardiovascular drugs	1,303 (21.86)	497 (18.15)	806 (25.01)
High frequency (≥10 administrations)	544 (7.56)	121 (3.92)	423 (10.28)
Low frequency (<10 administrations)	1,589 (22.08)	557 (18.07)	1,032 (25.09)
Did not take cardiovascular drugs	4,623 (64.24)	2,219 (71.98)	2,404 (58.45)
“Other” drugs (overall)	330 (4.59)	82 (2.66)	248 (6.03)
High risk “other” drugs	263 (3.66)	56 (1.82)	207 (5.04)
Low risk “other” drugs	56 (0.78)	21 (0.68)	35 (0.85)
High frequency (≥10 administrations)	203 (2.82)	40 (1.30)	163 (3.96)
Low frequency (<10 administrations)	220 (3.06)	53 (1.72)	167 (4.06)
Did not take “other” medications	6,866 (95.41)	3,001 (97.34)	3,865 (93.97)
**Skin tone (%)**			
Light	6,030 (83.80)	2,489 (80.73)	3,541 (86.09)
Medium	1,064 (14.79)	507 (16.45)	557 (13.54)
Dark	102 (1.42)	87 (2.82)	15 (0.36)
**Body part, lesion location by (%)**			
Head/neck	2,703 (37.56)	843 (27.34)	1860 (45.22)
Trunk	2,167 (30.11)	1262 (40.93)	905 (22.00)
Arms	1,039 (14.44)	374 (12.13)	665 (16.17)
Legs	905 (12.58)	407 (13.20)	498 (12.11)
Acral	281 (3.90)	121 (3.92)	160 (3.89)
Groin/buttocks	101 (1.40)	76 (2.47)	25 (0.61)

^1^Patient race was organized into the following categories: Caucasian/White, Black/African American, Asian, American Indian or Alaskan Native, Hawaiian or other Pacific Islander, Other, and Not Reported/Declined; 2 or more races and Unavailable were classified as Other and Not Reported/Declined, respectively.

^2^CCS groupings used were: history of chronic ulcer of skin, diseases of white blood cells, human immunodeficiency virus [HIV] infection, Hodgkin’s disease, non-Hodgkin’s lymphoma, infective arthritis and osteomyelitis, leukemias, Parkinson’s disease, rheumatologic diseases, skin and subcutaneous tissue infections, inflammatory condition of skin, systemic lupus erythematosus, other connective tissue disease, other sexually transmitted diseases, other hematologic diseases, other skin disorders.

^3^CPT codes to select for transplant procedures: liver (Liver Transplantation Procedure - 47133, 47135, 47140, 47141, 47152, 47143, 47144, 47145, 47146, 47147), lung/cardiac (Lung Transplantation Procedures - 32851, 32853. Heart/Lung Transplantations Procedure - 33945), bone marrow (Bone Marrow or Stem Cell Services/Procedures - 38232), kidney (Renal Transplantation Procedures - 50360, 50365, 50370).

^4^We selected the following medication groupers of interest based on their potential associations with skin cancer: immunosuppressants, corticosteroids, anti-hypertensives, anti-fungals, diuretics, antibiotics, anti-arrhythmics, anti-thrombotics, chemotherapy drugs, targeted therapy drugs (BRAF-inhibitors), immunotherapy drugs, and “other” medications of interest (azathioprine, tacrolimus, cellcept, hydroxychloroquine, and methyldopa). Medications were then further classified into one of the following three groups based on literature review: high risk of causing skin cancer, low risk of causing skin cancer or used to treat skin cancer, or no correlation with causing skin cancer; medications belonging to the last group were not included in training the model.

### Image selection and identification

This was a single-institution, retrospective study utilizing clinical and dermoscopy skin lesion images with institutional review board approval. We reviewed all patient encounters from patients of age 18 and older from 2013 to 2018 in the Department of Dermatology at Duke University with biopsies, and sorted images associated with the respective pathology reports into a database. Images were captured using smartphones or smart devices. Detailed information on how images were sorted, selected, and annotated for model development can be found in the Supplementary materials and Methods section as well as in a footnote in Figure 1. Image exclusion criteria are listed in a footnote in Figure 1. For the binary classification, malignant lesions were defined as melanoma, BCC, and actinic keratosis/Bowen’s disease. Benign lesions included melanocytic nevus, benign keratosis, dermatofibroma, vascular lesion, and others (including rash images).

### Demographics – Age at encounter, gender, race

Patient encounter and medical chart data were collected from Epic Clarity tables *via* Structured Query Language (SQL) queries of a consolidated database. Specific patient encounters and demographic data based on the encounter IDs associated with the previously labeled clinical and dermatoscope images was curated. A detailed list of patient race categories used can be found in a footnote in Table 1. Encounter of interest (EOI) is defined as the particular encounter when a skin biopsy was performed that informed the image-only and image and clinical models.

### Prior diagnoses and surgical history

Patient diagnoses were curated using International Classification of Diseases (ICD) 10 codes based on patient diagnoses present in the patient’s problem list in Epic. We then grouped the ICD codes of interest using the Clinical Classifications Software (CCS) method^1^ and indicated if a patient had a history of the disease *via* binary classification of a given CCS group. We curated procedural data using Common Procedural Terminolology (CPT) codes for procedures prior to EOI. Prior liver, lung/cardiac, bone marrow, and kidney transplants were noted. A detailed list of the CCS groupings used for history of comorbidities and CPT codes used for transplantation procedures can be found in a footnote in Table 1.

### Medications and grouper curation process

Two types of medication lists were curated: Medication administration record (MAR) medications, which are administered to the patient either in an inpatient or outpatient setting, and Medlist medications, which are present on a patient’s medication list in Epic and represent prescribed medications and patient-reported, typically in outpatient setting. Details on the process used to create medication groupers of interest can be found in the Supplementary materials and Methods section, and a detailed list of the medication groupers of interest can be found in a footnote in Table 1. Each patient and EOI was linked to MAR and Medlist medications, hence medication exposure data was highly accurate and quantitative. For MAR medications, medication administered could be tallied before EOI, providing an accurate quantitative value to inform the clinical model. We used a binary classification system to indicate if a patient was administered or had a history of taking a particular medication grouper class prior to the EOI.

### Office visits

Prior patient encounters in dermatology offices prior to EOI were tallied by selecting for encounters in departments containing either “DERM” or “MOHS”.

### Model development and statistical analysis

**FIGURE 2:**
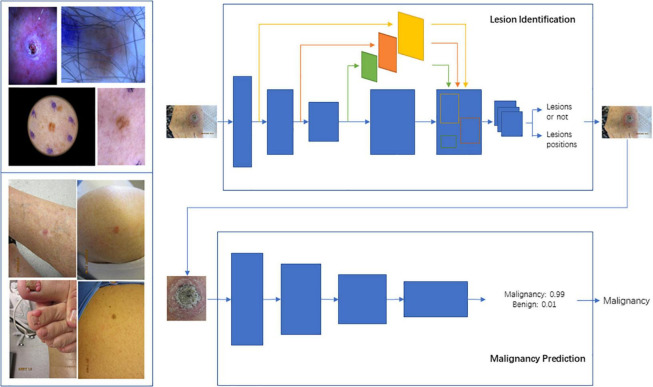
Schema for Lesion Identification and malignancy detection. Two-stage malignancy prediction and lesion identification Framework. **(Top left)** Examples of dermoscopy images. **(Bottom left)** Examples of wide-field images. **(Top right)** The lesion identification model estimates lesion locations (bounding boxes) from whole images (dermoscopy or wide-field) *via* a faster-RCNN architecture. **(Bottom right)** The malignancy prediction model specified *via* a ResNet-50 architecture predicts the likelihood that a lesion is malignant. The lesions identified by the lesion identification model are fed into the malignancy prediction model for end-to-end processing.

Detailed description of a two staged approach and methodology for development of an image-alone model, clinicodemographic data-alone model, and the combined image and data model are detailed in Figure 2 a complementary technical paper (arXiv:2104.02652). The image-alone and combined models were constructed as ResNet-50s, which are variation of CNNs (18, 19). The clinical data-alone model was built as a logistic regression model with standardized input and discrete (categorical) covariates encoded as one-hot vectors. Specifically regarding the combined image and data model, we used the malignancy classification model as the backbone while freezing all convolutional layers during training. Then, we concatenated the standardized input covariates and the global average-pooled convolutional feature maps and fed them through a fully connected layer with sigmoid activation that produces the likelihood of malignancy. The combined model was trained using an SGD optimizer for 30 epochs, with batch size 64, initial learning rate 0.001, momentum 0.9 and weight decay 1e-4. The learning rate was decayed using a half-period cosine function as in the malignancy classification model. Patients were split into training, testing, and validation sets based on patient IDs. There was no overlap with the datasets used for each phase of model development. We calculated the sensitivity, specificity, positive predictive value (PPV; i.e., precision), area under the curve (AUC) of the receiver operating characteristic (ROC) curve, and average precision (AP) for each model and selected a threshold of 0.5 to determine the accuracy and predictive abilities of the models. The most superior model was tested for accuracy against three board-certified dermatologists, who individually evaluated an independent test set of 488 images comprising of clinical and dermoscopy images and rated them as benign vs. malignant. They also reported their confidence level with each classification.

**FIGURE 3:**
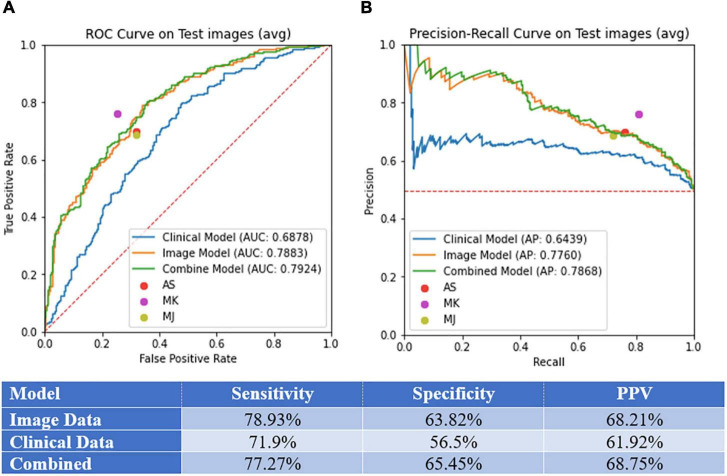
Model performance. **(A)** Area under the curve (AUC) of receiver operating curve (ROC) and precision-recall curves on test images. **(B)** Sensitivity, specificity, and PPV for the image-alone, clinical data-alone, and combined models.

### Dataset

#### Discovery dataset

To develop the model we consider a single institution, retrospective collection of skin lesion images taken with smartphones with and without dermoscopy from Duke University Medical Center patients of age 18 and older from 2013-2018. These data are collected under the approval of the Duke Institute for Health Innovation and each participant has provided written informed consent. The *discovery* dataset consists of 6,819 images from 3,853 patients with 7,196 manually annotated lesions, from which 4,113 (57%) lesions in 3,894 images are malignant. In terms of skin tone, 6,022 lesions (5,721 images) are light, 1,073 lesions (1,020 images) are medium and 101 lesions (96 images) are dark tone. Lesions were manually annotated as bounding boxes (ROIs) by a dermatology trained medical doctor (Dr. Kheterpal, MK) using a in-house annotation application. Diagnoses taken from the biopsy reports associated with the lesion images were designated as the ground truth (Malignant *vs.* Benign). Further, there are 589 (9%) dermoscopy images and 6,230 (91%) wide-field images. Table 1 shows detailed lesion type counts and proportions. The average area of the lesion is 307,699 (Q1-Q3: 9,192-184,900) pixels2 (roughly 554 × 554 pixels in size) while the average area of the images is 8’036,107 (3’145,728-12’000,000) pixels2 (roughly 2834 × 2834 pixels in size). We split the dataset, at the patient level, into 6,115 lesions (5,781 images) for training and 1,081 lesions (1,038 images) for validation. The validation set was used to optimize the model parameters, architecture and optimization parameters.

#### Clinical dataset

We also consider a subset of 4,130 images from 2,270 patients for which we also have demographic (age at encounter, sex and race), lesion characteristics (location and number of previous dermatology visits), comorbidities (history of chronic ulcer of skin, diseases of white blood cells, human immunodeficiency virus infection, Hodgkin’s disease, non- Hodgkin’s lymphoma, infective arthritis and osteomyelitis, leukemias, Parkinson’s disease, rheumatologic diseases, skin and subcutaneous tissue infections, inflammatory condition of skin, systemic lupus erythematosus, other connective tissue disease, other sexually transmitted diseases, other hematologic diseases, and other skin disorders) and skin-cancer-related medications (immunosuppressants, corticosteroids, antihypertensives, antifungals, diuretics, antibiotics, antiarrhythmics, antithrombotics, chemotherapy, targeted therapy, immunotherapy, and other), their risk (Low *vs.* High), and frequency of administration. Among these patients, 1,411 (2,537 images) are diagnosed as malignant and 859 (1,593 images) as benign. Similar to the discovery dataset, we split these data into 85% for training and the remaining 15% for validation.

#### ISIC2018

Provided that we have a smaller number of dermoscopy images, we also consider augmenting our discovery dataset with the ISIC2018 training dataset 24, 25 consisting of 10,015 dermoscopy images, from which 1,954 correspond to malignant lesions and 8,061 benign lesions. In the experiments, we also consider the ISIC2018 validation dataset to test the model with and without ISIC2018 augmentation.

## Results

### Comparison of model performance on binary classification

We selected a threshold of 0.5 to calculate the sensitivity, specificity, and PPV for each model, as standard practice. Sensitivity, specificity, PPV, AUC, and AP values for each model can be found in Figures 3A,B. The models demonstrated the following performance, respectively: sensitivity (image-alone: 78.9%, clinical data-alone: 71.9%, combined: 77.3%), specificity (image-alone: 63.8%, clinical data-alone: 56.5%, combined: 65.2%), PPV (image-alone: 68.2%, clinical data-alone: 61.9%, combined: 68.8%), AUC (image-alone: 78.8%, clinical data-alone: 68.8%, combined: 79.2%), AP (image-alone: 77.6%, clinical data-alone: 64.4%, combined: 78.7%). The image-alone and combined image and clinical data models had comparable sensitivity, specificity, PPV, AUC, and AP while the clinical data-alone model performed the worst in all metrics out of the three models. The addition of clinical data to the image-alone model surprisingly did not seem to improve the model’s performance significantly. In addition, the skin lesion image rather than clinical features of the patient with the skin lesion seem to have a greater importance in determining if the lesion is benign vs. malignant. For the clinical data-alone model, all of the clinicodemographic features except history of organ transplantation were found to be statistically significant in predicting skin malignancy (Table 2). However, certain features were the most significantly associated with a higher chance of malignancy. For example, patients who were older, identified as males, and had a greater number of previous dermatology visits prior to a given visit had a higher chance of having malignant skin lesions. This is concordant with national trends that show increasing incidence of melanoma, the only skin cancer reportable in the SEER database, with age and in males (6). In addition, patients with underlying skin conditions or have lesions that require frequent dermatology visits are more likely to have malignant lesions. Other features significantly associated with a higher chance of malignancy included having a comorbidity under the category of “HIV infection” or “Systemic lupus erythematosus and connective tissue disorders” and having a history of taking a high-risk “other” medication (azathioprine, tacrolimus, cellcept, hydroxychloroquine, or methyldopa). These findings could be due to the effects that autoimmune skin conditions and immunosuppressants have on inhibiting repair mechanisms that would normally be protective against skin malignancy (20). In contrast, certain features were found to be the most significantly associated with a lower chance of skin malignancy, such as having a history of taking a low-risk antibiotic medication, identifying as Asian or black/African American race, having a lesion located on the hands/feet or buttocks/groin region, and having a darker skin tone (Table 2). These findings could be due to the protective effects of a darker skin tone and areas of the body that have low UV light exposure on development of skin malignancy. One controversial result noted was a lower risk of malignancy in patients with a history of a prior cardiac or lung transplant. Typically these patients have a high degree of immunosuppression and thus these patients are typically in high risk category (21). However in our study, only 46 patients were included in this rare cohort, thus results were statistically non-signficant and may not be reflective of the true incidence. Bias may also be introduced by the clinical practice of routinely monitoring these patients despite no concerning lesions as part of routine post transplant surveillance as well as lower threshold for biopsing skin lesions, therefore favaoring benignity.

**TABLE 2:** Coefficients and associations for demographic and clinical features relevant for informing the combined image and clinical data model.

Feature	Coefficient	Correlation with malignancy
Older Age	0.700487	Positive
Male sex	0.149656	Positive
Comorbidity of HIV infection	0.139267	Positive
Comorbidity in the category “Systemic Lupus Erythematosus and Other Connective Tissue Disorders”	0.088971	Positive
Taking a low-risk antibiotic medication	–0.196025	Negative
Taking a high-risk medication in the “other” category	0.132797	Positive
Asian Race	–0.111828	Negative
Black or African American Race	–0.274017	Negative
History of prior cardiac or lung transplant	–0.258418	Negative
Higher number of previous visits to dermatology	0.175964	Positive
Part of the body the lesion was located	–0.115341	Negative
Darker skin tone	–0.065430	Negative

### Comparison of model performance on skin tone and race

Performance for each model on images representing light vs. medium/dark skin tones can be found in Table 3. Without any knowledge of patients’ gender, race, and ethnicity, 2 raters independently rated images as light skin tone based on consistency with Fitzpatrick type 1&2, medium skin tone consistent with Fitzpatrick type 3&4, and dark skin tone consistent with Fitzpatrick type 5&6. The image-alone and combined image and clinical data models performed significantly better on images classified as having light skin tones, with an AUC of 79.9% and 80.2%, respectively, compared to the clinical data-alone model, with an AUC of 67.3%. The image-alone and combined models also had marginal improvement in performance on images classified as having medium and dark skin tones, with an AUC of 75.6 and 76.3%, respectively, compared to the clinical data-alone model, with an AUC of 73.0%. Performance for each model on images from individuals identifying as white vs. non-white race can be found in Table 4. The image-alone and combined image and clinical data models performed better on images from patients who identified as a white race, with an AUC of 78.0 and 78.34%, respectively, compared to the clinical data-alone model, with an AUC of 67.3%. The image-alone and combined image and clinical data models also performed better on images from patients who identified as a non-white race, with an AUC of 89.6 and 89.6%, respectively, compared to the clinical data-alone model, with an AUC of 66.7%. These findings could further highlight the importance of image-related features over patient demographic features in predicting if a lesion is benign vs. malignant.

**TABLE 3:** Performance/AUC for each model on light vs. medium/dark skin tones on a small test dataset (*n* = 488).

	Light skin tone (*n* = 359)	Medium and dark skin tone (*n* = 129)	Total
Image model	0.7994	0.7563	0.7883
Clinical model	0.6729	0.7297	0.6878
Combined model	0.8018	0.7634	0.7924

**TABLE 4:** Performance/AUC for each model on white vs. non-white races on a small test dataset (*n* = 488).

	White race (*n* = 460)	Non-white race (*n* = 28)	Total
Image model	0.7799	0.8958	0.7883
Clinical model	0.6733	0.6667	0.6878
Combined model	0.7834	0.8958	0.7924

### Comparison of image-alone model against dermatologists

Three-board certified dermatologists classified a separate independent test set of 488 skin lesion images as benign vs. malignant against histological ground truth. The model correctly identified malignant lesions with an accuracy of 71.3% at a threshold of 0.5 against the three board-certified dermatologists who correctly identified malignant lesions with an accuracy of 77.9, 69.9, and 71.9%, respectively.

## Discussion

### Utility of real-world smartphone images

Early skin lesion classification models relied on high-quality clinical and dermoscopy images for proof of concept. Models trained on these high-quality images may have limited applications in primary care facilities and resource-limited rural settings. While there are challenges to consumer grade smartphone image quality, such as variability in image angles, lighting, distance from lesion of interest, and blurriness, they show promise to improve clinical workflows in primary care. Wide field images acquired by smartphones can be easily acquired during clinical workflow and therefore can democratize the process of dermatological care access by appropriate triage. Soenksen et al. demonstrated the utility of wide-field clinical images taken with smartphones for detection of “ugly duckling” suspicious pigmented lesions vs. non-suspicious lesions with 90.3% sensitivity (95% CI: 90.0-90.6) and 89.9% specificity (95% CI: 89.6-90.2) validated against three board-certified dermatologists (22). However, they used consensus of the dermatologist instead of more definitive histological ground truth. This is relevant as there is variability in the dermatologists’ accuracy and number needed to treat (NNT) metrics. NNT for true melanoma detection from pigmented lesion biopsies by dermatologists is 9.60 (95% CI: 6.97-13.41) by meta-analysis (23). This demonstrates the importance of using histopathological reports as ground truth rather than concordance with dermatologists for improved accuracy and comparability of model performance. Other models used multiple clinical images as input (16), while our model used clinical or dermoscopy images. Interestingly, the addition of dermoscopy images that were not paired with corresponding clinical images also significantly improved model performance (arXiv:2104.02652), demonstrating that the addition of paired or unpaired images may help improve future model performance. Our image-alone and combined models were trained and validated with wide-field clinical and dermoscopy images taken with smartphones based on histopathological ground truth and demonstrated encouraging model performance, sensitivity, and specificity outcomes to current literature (11–13). The usability of these models were further validated by comparison with dermatologists with variable levels of dermoscopy experience, showing comparable performance to dermatologists in both clinical and dermoscopy binary classification tasks.

### Demographics and clinical features

Early models have considered either only image data or clinical data in training algorithms to classify lesions as benign or malignant. Clinicians often make contextual diagnostic and management decisions while evaluating skin lesions to improve their accuracy and clinical-context improves diagnostic accuracy in pigmented lesions. However, this may be dependent on years of dermoscopy experience (12), largely disappearing in expert reviewers. Our combined model considered both image and clinical data to predict if a lesion is benign vs. malignant. We demonstrate that comprehensive EHR demographic and clinical data, while relevant to risk of malignancy as shown previously (12, 17), may not be critical for improving CNN-model performance in a subset of patients when compared with images for lesion classification. There are several possibilities why clinical context maybe less important in this setting. Specific lesion characteristics, such as color variegation and skin tone, may inform the model robustly to account for clinical context such as age, race, and ethnicity. Lesion features may be more significant in determining the malignant potential of a lesion than prior history including procedure and medication exposure. This differs from recent work done by Liu et al., who also integrated clinical data into their model and showed a 3% improvement in model performance; however, these results are challenging to interpret since it is unclear which clinical features were used to train their model as well as if this improvement in accuracy was for skin growths, rashes, or both (16). It should be noted that the current study was performed on lesions selected by dermatology providers for a biopsy, hence the results may not generalize to clinical settings such as primary care. Additionally, clinical-data models do not encompass additional high-risk features for skin cancer such as present or past chronic sun exposure, severe intermittent sun exposure, tanning booth exposure, and family history of skin cancer, although these data are not robust due to inconsistent and retrospective nature of capture as well as recall bias. The image model performing at par with the combination model implies that the infrastructure and resources needed to incorporate clinical data into the models may not be needed. Smaller clinical practices with fewer resources and busy workflows could still utilize this model as CDS *via* their smartphones without complex EHR integrations.

### Model performance based on skin tone and race

While fair skin tones were over-represented (Table 3), we demonstrate comparable AUC for the models based on light and medium/dark skin tones. While ethnicity bias in existing image datasets is explicitly noted, our study aims to stratify results based on a blinded assessment of skin tone independent of ethnicity. Adequate representation of skin variation is important for widespread use of CNN-based artificial intelligence (AI) models (24) and may not be dependent on ethnicity, which is more complex and important for clinical context but not image-data models in our study. The models also demonstrated comparable AUC based on white and non-white races, with improved performance on non-white race skin lesions for the image-alone and combined data models despite having a lower proportion of patients who identified as a non-white race. However, the applicability of these results may be limited as there were only 28 non-white race skin lesions and only 4 of these lesions were classified as malignant.

### Comparison of image-alone model against dermatologists

Our proof of concept model performed comparatively well against three board-certified dermatologists, demonstrating its utility as a classification tool that performs as well as current experts in the field. Before implementation in a clinical setting, however, it is important to validate model performance in a clinical workflow, testing a larger set of images with a larger set of dermatogists prior to deployment. This is important as our model was trained on images that utilized histopathological reports as ground truth while dermatologist consensus is often used as the ground truth in a real-world clinical setting, as many lesions are not biopsied.

### Limitations

Limitations of the study include a small discovery image dataset and a limited number of dermoscopy images. In particular, images with dark skin tone represented less than 2% of the images. While this may represent bias, skin cancers are more prevalent in light- and medium-skin tones. However, for a generalized CDS, it is important to incorporate images representing the large range of skin types and lesions encountered in clinical practice. As education efforts expand to represent more dark skin images of dermatologic conditions in educational materials (25), similar efforts should be pursued to develop and train generalizable CDS tools. Additionally, while the pure clinical model incorporates a comprehensive list of patient demographics, comorbidities, and medications and accounts for temporal association of this metadata with detection of lesions, it is not an exhaustive list as it does not include social determinants such as sun-exposure behavior (26), tanning bed usage, or smoking behavior (27): critical factors contributing to the increasing incidence of skin cancer. Metadata such as lesion symptoms (ex. bleeding or itching) (16) and evolution of lesions is also missing and should be incorporated in future studies. Interestingly, the addition of skin tone and location of the skin lesion of interest caused the performance of the clinical data-alone model to decline. This could be due to subjective labeling of the skin tone of each of the skin lesion images as light, medium, or dark rather than more standardized classification of skin tone used in other studies, such as the Fitzpatrick skin type classification scale (16). As for location of the skin lesion of interest, while one study found that most BCCs are located on the head, neck, and trunk (28), which is concurrent with our model, our model predicted a lower risk of malignancy for lesions on areas such as the extremities, which often have higher sun exposure. Finally, it should be noted that lesions included in this study were evaluated and selected for biopsies in dermatology clinics. If this model was to be utilized in a primary care setting, additional validation would be needed as pre-test probability of lesion detection is different among clinical settings (23).

## Data availability statement

The raw data supporting the conclusions of this article will be made available by the authors, without undue reservation.

## Ethics statement

The studies involving human participants were reviewed and approved by Duke Institutional Review Board. Written informed consent for participation was not required for this study in accordance with the national legislation and the institutional requirements.

## Author contributions

SW: software, data curation, writing – original draft, and writing – review and editing. WR: software, data curation, and writing – review and editing. MX: methodology, formal analysis, resources, data curation, and writing – review and editing. CP: data curation and writing – review and editing. MS: writing – review and editing. SB: resources, supervision, and project administration. RH and LC: methodology, formal analysis, and resources. MK: conceptualization, resources, investigation, data curation, writing – original draft, writing – review and editing, supervision, project administration, and funding acquisition. All authors contributed to the article and approved the submitted version.

## Footnotes

1
https://www.hcup-us.ahrq.gov/toolssoftware/ccs/ccs.jsp

## References

[1] Feng H Berk-Krauss J Feng P Stein J. Comparison of dermatologist density between urban and rural counties in the United States. *JAMA Dermatol.* (2018) 154:1265–71. 10.1001/jamadermatol.2018.3022 30193349 PMC6248119

[2] Resneck J Kimball AB. The dermatology workforce shortage. *J Am Acad Dermatol.* (2004) 50:50–4. 10.1016/j.jaad.2003.07.001 14699364

[3] Tsang MW Resneck JS. Even patients with changing moles face long dermatology appointment wait-times: a study of simulated patient calls to dermatologists. *J Am Acad Dermatol.* (2006) 55:54–8. 10.1016/j.jaad.2006.04.001 16781292

[4] Lowell BA Froelich CW Federman DG Kirsner RS. Dermatology in primary care: prevalence and patient disposition. *J Am Acad Dermatol.* (2001) 45:250–5. 10.1067/mjd.2001.114598 11464187

[5] Vincent G. *The Next Four Decades: The Older Population In The United States: 2010 To 2050.* Washington, DC: US Department of Commerce, Economics and Statistics Administration (2010).

[6] National Cancer Institute. *Cancer Stat Facts: Melanoma of the Skin.* Bethesda, MD: National Cancer Institute (2018).

[7] Wagner J Hall JD Ross RL Cameron D Sachdeva B Kansagara D Implementing risk stratification in primary care: challenges and strategies. *J Am Board Fam Med.* (2019) 32:585–95. 10.3122/jabfm.2019.04.180341 31300579

[8] Moreno G Tran H Chia AL Lim A Shumack S. Prospective study to assess general practitioners’ dermatological diagnostic skills in a referral setting. *Aust J Dermatol.* (2007) 48:77–82. 10.1111/j.1440-0960.2007.00340.x 17535192

[9] Rismiller K Cartron AM Trinidad JCL. Inpatient teledermatology during the COVID-19 pandemic. *J Dermatolog Treat.* (2020) 31:441–3. 10.1080/09546634.2020.1762843 32364809

[10] Pulsipher KJ Presley CL Rundle CW Rietcheck HR Millitelo M Dellavalle RP. Teledermatology application use in the COVID-19 era. *Dermatol Online J.* (2020) 26:5. 10.5070/D32612051350 33423415

[11] Esteva A Kuprel B Novoa RA Ko J Swetter SM Blau HM Dermatologist-level classification of skin cancer with deep neural networks. *Nature.* (2017) 542:115–8. 10.1038/nature21056 28117445 PMC8382232

[12] Haenssle HA Fink C Schneiderbauer R Toberer F Buhl T Blum A Man against machine: diagnostic performance of a deep learning convolutional neural network for dermoscopic melanoma recognition in comparison to 58 dermatologists. *Ann Oncol.* (2018) 29:1836–42. 29846502 10.1093/annonc/mdy166

[13] Safran T Viezel-Mathieu A Corban J Kanevsky A Thibaudeau S Kanevsky J. Machine learning and melanoma: the future of screening. *J Am Acad Dermatol.* (2018) 78:620–1. 10.1016/j.jaad.2017.09.055 28989109

[14] Han SS Park I Eun Chang S Lim W Kim MS Park GH Augmented intelligence dermatology: deep neural networks empower medical professionals in diagnosing skin cancer and predicting treatment options for 134 skin disorders. *J Invest Dermatol.* (2020) 140:1753–61. 10.1016/j.jid.2020.01.019 32243882

[15] Brinker TJ Hekler A Enk AH Klode J Hauschild A Berking C A convolutional neural network trained with dermoscopic images performed on par with 145 dermatologists in a clinical melanoma image classification task. *Eur J Cancer.* (2019) 111:148–54. 30852421 10.1016/j.ejca.2019.02.005

[16] Liu Y Jain A Eng C Way DH Lee K Bui P A deep learning system for differential diagnosis of skin diseases. *Nat Med.* (2020) 26:900–8. 10.1038/s41591-020-0842-3 32424212

[17] Wang HH Wang YH Liang CW Li YC. Assessment of deep learning using nonimaging information and sequential medical records to develop a prediction model for nonmelanoma skin cancer. *JAMA Dermatol.* (2019) 155:1277–83. 10.1001/jamadermatol.2019.2335 31483437 PMC6727683

[18] He K Zhang X Ren S Sun J. Deep Residual Learning for Image Recognition. *2016 IEEE Conference on Computer Vision and Pattern Recognition (CVPR).* Piscataway, NJ: IEEE (2016). 10.1109/CVPR.2016.90

[19] Ren S He K Girshick R Sun J. Faster R-CNN: towards real-time object detection with region proposal networks. *Proceedings of the 28th International Conference on Neural Information Processing Systems - Volume 1.* Montreal, QC: MIT Press (2015). p. 91–9.

[20] Gerlini G Romagnoli P Pimpinelli N. Skin cancer and immunosuppression. *Crit Rev Oncol Hematol.* (2005) 56:127–36. 10.1016/j.critrevonc.2004.11.011 15978830

[21] Collins L Quinn A Stasko T. Skin cancer and immunosuppression. *Dermatol Clin.* (2019) 37:83–94. 10.1016/j.det.2018.07.009 30466691

[22] Soenksen LR Kassis T Conover ST Marti-Fuster B Birkenfeld JS Tucker-Schwartz J Using deep learning for dermatologist-level detection of suspicious pigmented skin lesions from wide-field images. *Sci Transl Med.* (2021) 13:eabb3652. 10.1126/scitranslmed.abb3652 33597262

[23] Petty AJ Ackerson B Garza R Peterson M Liu B Green C Meta-analysis of number needed to treat for diagnosis of melanoma by clinical setting. *J Am Acad Dermatol.* (2020) 82:1158–65. 10.1016/j.jaad.2019.12.063 31931085 PMC7167347

[24] Adamson AS Smith A. Machine learning and healthcare disparities in dermatology. *JAMA Dermatol.* (2018) 154:1247. 10.1001/jamadermatol.2018.2348 30073260

[25] Alvarado SM Feng H. Representation of dark skin images of common dermatologic conditions in educational resources: a cross-sectional analysis. *J Am Acad Dermatol.* (2021) 84:1427–31. 10.1016/j.jaad.2020.06.041 32565205

[26] Zak-Prelich M Narbutt J Sysa-Jedrzejowska A. Environmental risk factors predisposing to the development of basal cell carcinoma. *Dermatol Surg.* (2004) 30(Pt. 2):248–52. 10.1111/j.1524-4725.2004.30089.x 14871217

[27] Dusingize JC Olsen CM Pandeya NP Subramaniam P Thompson BS Neale RE Cigarette smoking and the risks of basal cell carcinoma and squamous cell carcinoma. *J Invest Dermatol.* (2017) 137:1700–8. 10.1016/j.jid.2017.03.027 28414022

[28] Kasumagic-Halilovic E Hasic M Ovcina-Kurtovic NA. Clinical study of basal cell carcinoma. *Med Arch.* (2019) 73:394–8. 10.5455/medarh.2019.73.394-398 32082007 PMC7007603

