# Supplementary material for: Use of convolutional neural networks in skin lesion analysis using real world image and non-image data
Source: Front Med (Lausanne). 2022 Oct 19;9:946937. doi: 10.3389/fmed.2022.946937 (PMC9629864; doi:10.3389/fmed.2022.946937)
Supplement: Supplementary file 1 [file Data_Sheet_1.DOCX]

**SUPPLEMENTARY MATERIAL AND METHODS**

**Image Selection and Identification**

Images were sorted in random order in the database and the subset of lesion images were selected and annotated using bounding boxes for area of interest and labelled according to the following criteria: image source (dermoscopy or nondermoscopy), lesion type per pathology diagnosis (melanoma, melanocytic nevus, BCC, actinic keratosis/Bowen’s disease [intraepithelial carcinoma]), benign keratosis [solar lentigo/seborrheic keratosis/lichen planus-like keratosis], dermatofibroma, vascular lesion, other), lesion location (head/neck, trunk, arms, legs, acral, groin/buttocks), hair (yes or no), skin color (light, medium, dark).

**Creation of Medication Groupers**

We grouped medications by Anatomic Therapeutic Chemical (ATC) classes according to RxNorm, which is a “normalized naming system [produced by the National Library of Medicine] for generic and branded drugs; and a tool for supporting semantic interoperation between drug terminologies and pharmacy knowledge base systems”(27). We ran comprehensive lists containing Duke MAR and Medlist medications through RxNorm, producing JavaScript Object Notation (JSON) files containing medications linked to their respective ATC classes; Duke MAR and Medlist medications that were not represented in RxNorm were manually assigned ATC classes according to their active component. ATC classes were then further sorted into medication “groupers” that represent broader medication classes.
